# Effects of multicultural piano education on children's psychosocial wellbeing: the roles of cross-cultural musical competence and cultural identity

**DOI:** 10.3389/fpsyg.2026.1942999

**Published:** 2026-09-18

**Authors:** Qi Yu, Panghua Tian

**Affiliations:** 1 College of Art, Shaanxi University of Technology, Hanzhong, Shaanxi, China; 2 College of Art and Design, Shaanxi Institute of Technology, Xi'an, Shaanxi, China

**Keywords:** children, controlled intervention, cross-cultural musical competence, cultural identity, emotional regulation, multicultural piano education, music education, psychosocial wellbeing

## Abstract

**Introduction:**

Children's psychosocial wellbeing is closely linked to emotional regulation, social competence, self-esteem, resilience, and cultural identity. Traditional piano instruction commonly prioritizes technical skills and Western classical repertoire, leaving its psychological and cross-cultural potential underexplored. This study examined whether a structured multicultural piano education programme could enhance psychosocial wellbeing in children aged 6–12 and whether cross-cultural musical competence and cultural identity mediated these effects.

**Methods:**

A 12-week stratified controlled intervention included 320 children, allocated to a multicultural piano education group (*n* = 160) or a conventional piano education group (*n* = 160). The experimental group received two 90-min instructor-led classes per week plus approximately 30 min of daily practice, integrating Chinese ethnic and traditional piano works with music from Southeast Asia, Europe, Africa, and Latin America. Assessments were conducted at baseline (T0), week 6 (T1), post-intervention at week 12 (T2), and a 3-month follow-up (T3). Outcomes included emotion regulation, SCARED anxiety, social competence, loneliness, self-esteem, psychological resilience, heart rate variability, cross-cultural musical competence, and cultural identity. Repeated-measures analyses, structural equation modeling, and bootstrap mediation analyses were performed.

**Results:**

Compared with conventional piano education, multicultural piano education produced more favorable changes in emotion regulation, anxiety, social competence, loneliness, self-esteem, psychological resilience, and heart rate variability. It also improved cross-cultural musical competence and cultural identity. Mediation analyses indicated that changes in cross-cultural musical competence and cultural identity partially accounted for the association between the intervention and the model-level psychosocial outcome construct.

**Discussion:**

Multicultural piano education may provide a practical educational approach that integrates aesthetic learning, intercultural awareness, and psychosocial development in children. The findings support the value of embedding culturally diverse musical content and structured cross-cultural learning activities within piano education.

## 1 Introduction

Psychosocial wellbeing in childhood refers to a child's ability to manage emotions, build good relationships, have a positive self-concept, handle changes in life, and participate actively in family, school and community life (Asher et al., 1984; Blasco-Magraner et al., 2021; Gross and John, 2003). The 6–12-year-old stage is relatively critical because children are starting to move away from family-centered life and have been exposed to various peer groups, formal schooling and higher social expectations. Difficulties that occur at this time, such as anxiety, loneliness, low self-esteem, emotional dysregulation and reduced social skills, can harm school adaptation and further psychological development. As a result, an increasing number of preventive measures have been introduced into the daily educational life of children without being labeled as psychological disabilities.

Arts-based education can provide a suitable environment to help children develop psychologically and socially (Blasco-Magraner et al., 2021; Hallam, 2010). Music activities involve multiple senses simultaneously, such as hearing, movement, attention, memory and emotion; they do not require verbal explanations or direct discussion of emotional problems. Active musical participation can also provide children with a sense of achievement, learn to handle mistakes, develop continuous attention, and express emotions in an organized way without words. Previous research has proposed that long-term music education can help children develop all-round abilities in thought, life and society; however, the degree and consistency of these effects vary among different study designs and outcomes (Hallam, 2010). More recent longitudinal and experimental studies have shown potential improvements in executive function, auditory processing and self-regulation after organized music education (Jaschke et al., 2018; Moreno et al., 2011; Nan et al., 2018). However, reviews of this field have pointed out that transfer effects should not be assumed, and strictly designed controlled experiments are needed to distinguish the effects of music education from maturation, teacher attention, family resources, and participation in other enrichment activities.

Music may influence psychosocial wellbeing through several complementary processes (Blasco-Magraner et al., 2021; Dingle et al., 2016; Hallam, 2010). First, musical performance requires children to regulate their own arousal, control frustration and modify expressive behavior, thus providing repeated opportunities for learning emotional regulation. Music is also often used by young people to keep, strengthen, change or explore their emotions (Saarikallio and Erkkilä, 2007). Second, after successfully learning progressively difficult music tasks, one will feel more capable and confident. According to the theory of self-determination, educational activities are more likely to promote the psychological needs for competence, autonomy and relatedness, and thus enhance wellbeing (Ryan and Deci, 2000). Thirdly, the group music activity can promote communication, joint attention, mutual responsiveness and socialization. Experiments have shown that joint music-making can increase cooperative and helping behavior in young children, and extended periods of group musical interaction have been associated with improvements in empathy (Kirschner and Tomasello, 2010; Rabinowitch et al., 2013).

Piano education offers a particular kind of environment for studying these processes. Learning the piano is a combined process of listening discrimination, visual notation, two-handed motor skills, memory, continuous attention, error correction, and emotional expression. The learner repeatedly compares the target and actual sounds, responds to corrective feedback, and perseveres despite temporary performance difficulties. These characteristics can foster self-regulation and emotional resilience in students of the positive-and-supportive class. Piano performance can also be used as a medium for children to express emotions such as tension, calmness, excitement, sadness and happiness without needing words. Experimental research on Mandarin-speaking children has found that piano training can enhance pitch-related auditory processing and some aspects of speech perception; thus, it is thought that piano learning may affect perceptual abilities other than technical skills (Nan et al., 2018). Most existing research on piano training has focused on cognitive, linguistic, auditory or motor results and neglected all-encompassing psychosocial health.

Another deficiency of traditional piano teaching is that it often focuses too much on technical perfection, graded examinations, and a small number of Western classical works. Western classical music is an excellent art form; however, a curriculum that focuses solely on techniques and culture may not provide children with any experience of the social values, historical background or other cultures that music carries. If children are taught to reproduce musical notes without knowing the cultural stories behind the works they learn, then piano learning will be divorced from their sense of self and cultural inheritance. A multi-cultural mode does not need to replace Western classical music. It will, in fact, add to the curriculum by presenting local, ethnic, national and foreign musical cultures in combination.

Multicultural music education introduces learners to musical systems, expressive conventions, instruments, rhythms, tonal organizations, stories and performance practices of different cultural communities (Abril, 2006, 2013; Cain, 2015). Such education can help children learn that the meaning of music is culturally relative and not universal or culture-free. Exposure is not sufficient on its own. If there is no context of culture or life experience, isolated segments labeled as “world music” may appear in a stereotypical way after comparison. Effective multicultural music education therefore needs to use culturally responsive teaching to connect the musical content with children's previous experiences, encourage critical comparison, and avoid presenting cultures as fixed or exotic. Music educators are willing to promote cultural diversity but do not know how to integrate the cultures of different countries into their teaching practices (Cain, 2015).

### 1.1 Cultural identity and cross-cultural musical competence

Culture identity is likely to promote the psychological health of multicultural music education students (Benet-Martínez and Haritatos, 2005; Birmaher et al., 1997; Blasco-Magraner et al., 2021; Bortz et al., 2025; Cain, 2015; Connor and Davidson, 2003; Dingle et al., 2016; Fu and Tu, 2023; Gerry et al., 2012; Gross and John, 2003; Guan et al., 2023; Phinney, 1992). Culture is a set of beliefs, practices and other forms that a group of people share. In childhood, musical experiences can help to form a sense of social, ethnic and musical identity by connecting songs and instrumental works with language, collective memory, rituals, places, family stories and community values (Guan et al., 2023; Phinney, 1992). Local music culture can help the children develop their sense of identity and belonging. At the same time, an extremely convenient meeting place for people all over the world can inspire people to be curious and learn new things. The above are not in opposition. A good multicultural curriculum can give people a sense of belonging in their own culture and motivate them to learn about other cultures with enthusiasm.

At present, the three components of cultural identity in this paper are introduced. Local cultural identification is a sense of belonging to one's hometown and country among children, interest in traditional music, and a feeling of cultural confidence. Acceptance of other cultures is to be open to different cultures, wanting to experience various music from different countries, and reducing cultural prejudice. Bicultural or multicultural integration is the capacity to view local and foreign cultures as compatible origins of musical and life meaning rather than as opposed ones. Multidimensional Conceptualization is required because strong cultural identity does not automatically lead to cross-cultural openness, and contact with foreign cultures does not necessarily reduce the sense of local culture.

Based on the above, research has begun to explore the contribution of music education to cultural identity. A culturally responsive music intervention in a multiethnic Chinese school showed improvements in several areas of students‘ ethnic identity, and qualitative data indicated that ethnic repertoire, native languages, local teachers and group musical activities were effective (Guan et al., 2023). Research on Chinese university students has also examined the relationship between national music education and subjective wellbeing, self-esteem and national identity, but the observed effects were not consistent across all outcome variables (Fu and Tu, 2023). Based on the above research, although culturally specific music education may impact individuals' sense of identity and quality of life, the degree of this impact also depends on various factors such as age, curriculum design, initial psychological state, etc. Evidence involving primary-school-aged children, multicultural piano teaching, objective physiological indices, and long-term follow-up is still lacking.

A second possible reason is cross-cultural musical literacy. Cross-cultural musical competence in this study does not refer only to factual knowledge of the place of origin for music. It refers to the ability at various levels to perceive, create, express, evaluate, and integrate music from all over the world creatively. Auditory features of a place include the recognition of distinctive rhythms, modes, tonal systems, and instruments. Its performance dimension refers to the capacity for replication of style in rhythm, dynamics and expression. The reflective mode is to compare and contrast all kinds of music to associate them with their own cultures. Creativity is the ability to use musical elements from many different cultures in improvisation or composition in an original way, not just to add ornaments.

Cross-cultural musical literacy can boost people's self-esteem and motivation in life by enhancing their sense of self-efficacy and optimism. Children who can recognize and perform many different types of music are more used to uncertainty and do not find unfamiliar cultural expressions threatening. A good cross-cultural performance can also offer real-life examples of how to understand different cultures through practice and communication. At the same time, the children's sense of cultural identity may also affect their learning. Children who have a secure and inclusive cultural identity are likely to be more curious about other musical cultures; those who feel that culture is different may be fearful and avoid learning. Cross-cultural musical competence and cultural identity are likely to work together to some extent rather than being independent developments.

Although these paths have theoretical value, there are still some deficiencies in the literature. First, many studies on general music participation do not specify the cultural composition, pedagogical structure or interactive elements of the curriculum. Second, psychosocial outcomes are frequently measured by a single self-report scale, and behavior or physiology is not often incorporated. Third, relatively few studies have examined both the direct intervention effects and the mechanisms of influence for music education on wellbeing. Fourth, research on culturally responsive music education has primarily focused on attitudes, identity or educational practices, and studies of music and mental health have often omitted cultural content. Therefore, the connections among multicultural music learning, cross-cultural musical competence, cultural identity and children's psychosocial wellbeing have not been fully explored.

### 1.2 The present study

Given the above deficiencies, the present study will implement a standardized 12-week multicultural piano education programme for children aged 6–12. The school will teach Chinese ethnic and traditional piano works, as well as pieces from Southeast Asia, Europe, Africa and Latin America. All classes have cultural stories, piano performance teaching, comparative listening, and cooperative music creation. The intervention group was compared with the control group of traditional piano classes that did not include regular multicultural content. At baseline, during the intervention, immediately after the intervention, and at a 3-month follow-up, psychosocial wellbeing, cross-cultural musical competence, cultural identity, behavioral indicators and heart rate variability were all measured.

First of all, it was hoped that multicultural piano education would lead to better changes in children's psychosocial scale scores and physiological regulation indicators than in traditional piano education. Second, we would like to know if the intervention has promoted cross-cultural musical literacy and cultural identity. The third is to determine whether changes in cross-cultural musical competence and cultural identity are responsible for the association between the intervention and psychosocial outcomes. It was assumed that:

1. *H1*: Children in multicultural piano education will show better changes in emotion regulation scores, SCARED anxiety scores, social competence, loneliness, self-esteem, psychological resilience and heart-rate variability compared with children in traditional piano education.
2. *H2:* Children in the multicultural piano education group will show more significant improvements in cross-cultural auditory discrimination, culturally appropriate performance, comparative musical understanding, multicultural creativity, local cultural identification, acceptance of other cultures, and multicultural integration.
3. *H3:* Changes in cross-cultural musical competence and cultural identity will partially mediate the relationship between multicultural piano education and the model-level psychosocial outcome construct used in the exploratory mediation analysis.
4. *H4:* An exploratory serial indirect pathway will be observed, in which multicultural piano education enhances cross-cultural musical competence, which is associated with cultural identity, and finally, with the model-level psychosocial outcome construct.
5. *H5:* Baseline cultural identity may moderate the effect of intervention, and thus the extent of psychological and intercultural change will vary according to children's initial level of local cultural identification, openness to other cultures, and multicultural integration.

## 2 Materials and methods

### 2.1 Study design and participants

This study had a 12-week stratified controlled intervention design. A total of 320 children aged 6–12 years were recruited from primary schools and community arts education institutions, and divided into a multicultural piano education group or a conventional piano education group, with 160 children in each group. The average age of the experimental group and the control group was 9.14 ± 1.82 years and 9.08 ± 1.79 years, respectively. Boys accounted for 51.3 and 50.0% of the two groups, respectively, and 58.8 and 56.9% of the participants were from urban areas. Stratification was based on age, sex and urban-rural location to improve the comparability of the baseline. The basic data are as follows: Table 1.

**Table 1:** Baseline characteristics of participants.

Variable	Experimental group (*n* = 160)	Control group (*n* = 160)	*p*
Age, years	9.14 ± 1.82	9.08 ± 1.79	0.766
Boys, *n* (%)	82 (51.3)	80 (50.0)	0.823
Urban residence, *n* (%)	94 (58.8)	91 (56.9)	0.732
Previous piano practice, min/week	21.6 ± 15.4	22.3 ± 16.1	0.691
SCARED total score	26.10 ± 7.42	25.82 ± 7.35	0.735
Social competence score	3.12 ± 0.49	3.10 ± 0.51	0.721
Cultural identity score	4.08 ± 0.62	4.09 ± 0.60	0.884
Cross-cultural music competence	3.54 ± 0.71	3.57 ± 0.69	0.700

Means and standard deviations are presented as mean ± standard error.

Eligible participants had no regular piano lessons and had not practiced in over a month. Children with hearing loss, hand-movement disorders, diagnosed severe psychiatric or developmental disorders, recent psychological treatment, or extensive overseas cultural exposure were excluded. Written informed consent was obtained from the legal guardians of all the participants.

Measurements were taken at baseline before the intervention (T0), at 6 weeks (T1), immediately after the intervention at 12 weeks (T2), and 3 months after the intervention (T3). T3 follow-up mainly focused on psychosocial well-being and cultural identity.

### 2.2 Intervention

The children in the experimental group participated in a standardized multicultural piano education programme for 12 weeks, with two 90-min classes per week and about 30 min of daily homework. The general intervention structure is as follows: Figure 1.

**Figure 1:**
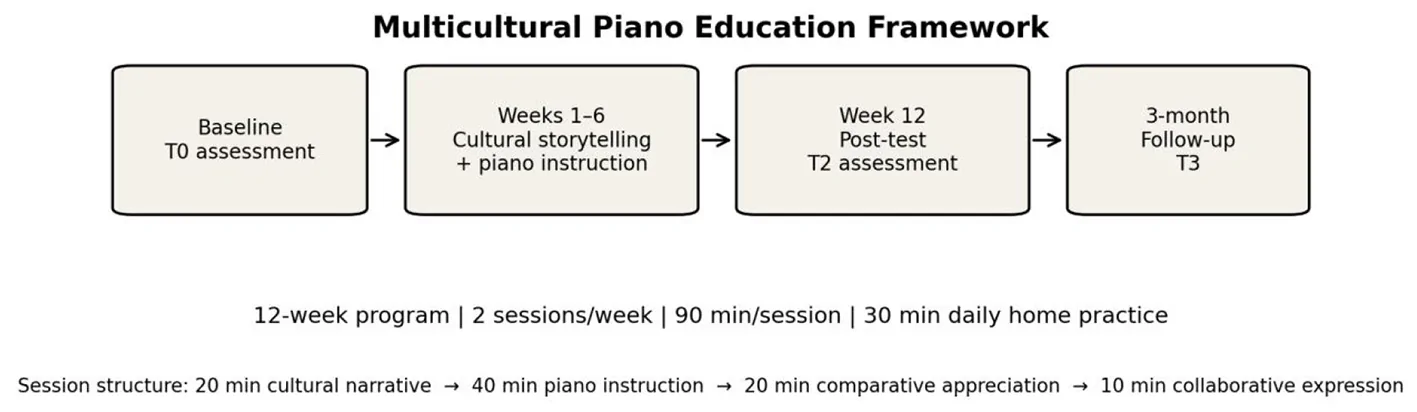
Structure and schedule of the multicultural piano education intervention.

The repertoire included about 50% of Chinese traditional and ethnic piano works, and the other 50% were culturally diverse works from Southeast Asia, Europe, Africa and Latin America. Repertoire selection corresponded to the technical level and developmental stage of each age group. Each class had a 20-min introduction to the culture, followed by 40 min of piano teaching, then a 20-min comparison of music, and finally a 10-min joint musical creation.

Teach different contents for different age groups. Children aged 6–8 years old completed simplified performance, rhythm imitation and visual cultural-expression activities. Children aged 9–12 completed short musical works, improvisation, group performances and culture-themed creative tasks.

Because the teaching content was developmentally adjusted, the intervention is reported below at the level that has been consistently documented throughout the study: cultural source, age-specific learning task, lesson component, and intended competency. This form offers a replicable account of the curriculum framework, but not all children have completed the same fixed-title repertoire (Table 2).

**Table 2:** Representative multicultural curriculum framework by cultural source and age group.

Repertoire/cultural source category	6–8 years: representative learning tasks	9–12 years: representative learning tasks	Primary instructional focus
Chinese traditional and ethnic piano works	Simplified performance; rhythm imitation; visual/cultural storytelling activities	Short works; stylistic interpretation; improvisation; culture-themed creative tasks	Local cultural identification; stylistic awareness; connection between musical features and cultural narratives
Southeast Asian musical traditions	Guided listening; rhythm/timbre recognition; short comparative activities	Comparative listening and performance; discussion of modes, timbres, and cultural context	Auditory discrimination; intercultural recognition; respectful comparison
European musical traditions	Age-appropriate performance and listening; contrast with Chinese repertoire	Performance, stylistic comparison, and reflective discussion	Technical control; stylistic comparison; recognition of culturally situated conventions
African musical traditions	Rhythm imitation; call-and-response style group activities; collaborative expression	Rhythmic comparison; group performance; improvisational tasks	Rhythmic diversity; cooperation; ensemble responsiveness
Latin American musical traditions	Rhythmic movement/imitation; simplified expressive performance	Dance-rhythm comparison; performance; improvisation and creative transfer	Expressive diversity; rhythmic flexibility; creative cross-cultural application

The archived report has preserved repertoire categories and age-differentiated learning tasks, but not a title-level inventory of all musical works; no unverified piece titles have been added retrospectively.

The control group was also in piano classes at the same time and place. Teaching focused on piano technique, music reading, rhythmic accuracy and Western classical children's repertoire without systematic multicultural explanation or cross-cultural comparison.

Teachers in both groups had similar qualifications and used the same classrooms, instruments and teaching schedules.

### 2.3 Outcome measures

#### 2.3.1 Psychosocial wellbeing

The main psychosocial outcomes were represented by continuous scores of emotion regulation, anxiety-related experiences, social competence, loneliness, self-esteem and psychological resilience, and resting heart rate variability (HRV) served as a supporting physiological indicator. Only the dimensionally reduced questionnaire scores were used; no psychiatric diagnoses or clinical cases were recorded. Therefore, a lower SCARED and loneliness score and a higher score in adaptive psychosocial measures were considered to be more favorable scale profiles rather than indications of psychopathology.

The 10-item Emotion Regulation Questionnaire for Children and Adolescents (ERQ-CA; Gullone and Taffe, 2012) was used to measure emotional regulation, and it includes cognitive reappraisal and expressive suppression, and was first developed to assess people aged 10–18 years old. The Screen for Child Anxiety Related Emotional Disorders (SCARED; Birmaher et al., 1997) was used to assess the level of anxiety experienced by children and parents. Rosenberg Self-Esteem Scale (RSES, 10 items) and Connor-Davidson Resilience Scale (CD-RISC, 25 items) were used to measure self-esteem and psychological resilience, respectively (Connor and Davidson, 2003; Schmitt and Allik, 2005). Social competence and loneliness were assessed with the child social-skills and loneliness scales. Since the sample included children younger than the upper limit for several instrument validation, all questionnaire results were used as continuous research variables; no clinical cut-off, diagnosis or treatment-response classification was performed.

The archived study documentation does not allow for reliable verification of the administration mode used for children aged 6–8 years. Therefore, the revision does not retrospectively assert parent-proxy reporting or researcher-assisted completion. Therefore, this uncertainty is regarded as a measurement error, and the results for the youngest age group are conservatively interpreted at the group level. Item-level raw response files and administration logs were also not available in the manuscript archive; therefore, sample-specific Cronbach's α/ω values that cannot be reliably recalculated are neither manufactured nor back-calculated from summary scores (Table 3).

**Table 3:** Measurement framework, scoring interpretation and developmental precautions.

Measure/ indicator	Construct used in this study	Reference format/role	Interpretation in this study	Developmental caution
ERQ-CA (Gullone and Taffe, 2012)	Emotion regulation	10-item self-report; cognitive reappraisal and expressive suppression; originally evaluated at ages 10–18	Continuous scale scores; higher adaptive-strategy scores interpreted as more favorable regulation profile	Youngest participants are below the principal validation age range; sample-specific α/ω not recoverable from archived item-level data.
SCARED (Birmaher et al., 1997)	Anxiety-related experiences	Child/parent screening instrument for anxiety-related experiences	Continuous total score; lower score interpreted as a lower anxiety-related rating, not remission of a disorder	Used dimensionally; no diagnostic cut-off applied. Administration mode for ages 6–8 cannot be verified from archived records.
RSES (Connor and Davidson, 2003)	Global self-esteem	10-item global self-esteem measure	Continuous score; higher score interpreted as greater self-esteem	Age-related interpretation is cautious; sample-specific α/ω not recoverable from archived item-level data.
CD-RISC (Schmitt and Allik, 2005)	Psychological resilience	Original 25-item scale; items rated 0–4	Continuous score; higher score interpreted as greater reported resilience	Original instrument was developed outside the youngest age range; interpreted as a dimensional research score.
Resting HRV (Shaffer and Ginsberg, 2017; Task Force of the European Society of Cardiology and the North American Society of Pacing and Electrophysiology, 1996)	Autonomic regulation	5-min resting recording; SDNN and RMSSD reported	Supportive physiological indicator, interpreted with questionnaire/behavioral outcomes	Supportive physiological indicator; device-level acquisition details were not fully retained in the archive.
37-item cultural identity measure	Local cultural identification; acceptance of other cultures; bicultural integration	Study-specific multidimensional questionnaire: 15 + 12 + 10 items	Domain scores analyzed as continuous outcomes	Study-specific measure; item-level provenance, response anchors, and subscale reliability were not retained in sufficient detail for reconstruction.

Measures are listed according to the information stored in the study archive. Where age-specific administration details or item-level reliability data could not be verified, the manuscript states this explicitly instead of reconstructing procedures or coefficients post hoc.

Heart-rate variability was recorded for 5 min at rest, and the main physiological indices used were SDNN and RMSSD (Shaffer and Ginsberg, 2017; Task Force of the European Society of Cardiology and the North American Society of Pacing and Electrophysiology, 1996). HRV was taken as a supporting physiological indicator rather than an independent index of emotional regulation because it is affected by age, exercise status, breathing and sleep, posture, etc. Classroom behavior includes active participation, peer help, cooperation in communication and coordination of performance, and is recorded by video.

The archived report shows that a 5-min resting HRV was obtained and SDNN and RMSSD were used; however, there is no other technical information available in the report, such as device model, sampling rate, time-of-day standardization, pre-assessment activity restrictions, respiration control, or artifact correction. Therefore, these details have not been reconstructed afterwards. HRV findings are considered supportive and exploratory of emotional regulation, but not direct evidence of it.

Video-derived classroom behaviors were secondary observational indicators and not the main endpoints. The archived documentation lists the target behaviors but does not contain the full coder-training, blinding or inter-rater reliability protocol. Therefore, the above observations are considered descriptive only and not used to establish the primary intervention effect.

#### 2.3.2 Cross-cultural musical competence

Cross-cultural musical competence was evaluated in terms of listening, performance, reflection, and creation.

Auditory Tasks include recognizing culturally specific rhythms, musical modes and instrumental colors. Two blind piano teachers independently scored the performance tasks according to the criteria of rhythmic accuracy, stylistic expression, emotional interpretation and cultural appropriateness.

Children also had group oral comparisons and improvisations to learn about the similarities and differences in cultures, express their views on what music signifies, and incorporate various elements from different musical cultures.

#### 2.3.3 Cultural identity

A 37-item research-specific multiple-choice question sheet was developed to investigate cultural identity, and the three parts were: recognition of local culture (15 items), acceptance of other cultures (12 items), and bicultural integration (10 items). A high domain score indicates a rich local culture and more cross-cultural openness and integration. As the archived materials have not established this questionnaire as a fully externally validated standardized scale, it has been reported as a study-specific measure and interpreted at the domain-score level.

The archived manuscript does not store item-level provenance, response anchors or subscale reliability estimates in detail enough for independent reconstruction. These properties are therefore not retrospectively invented. The cultural-identity findings are interpreted as extensions of the original study area, and it is acknowledged that independent psychometric verification has not been conducted.

Extra behavioral indicators are the time spent listening to various cultures' music and how often a person independently finds multicultural music.

### 2.4 Intervention fidelity and safety

Record the attendance and teaching fidelity after each class. Teachers recorded the teaching content covered, participants' participation, homework completion, emotions and deviations from the intervention plan.

Possible Risks include temporary emotional upset, hand fatigue, slight physical discomfort and interpersonal conflicts during group activities. The activities were suspended because the children were upset. Children were permitted to rest, withdraw from particular tests or stop taking part without penalty.

All Questionnaires, physiological data and recordings were coded anonymously. Only research-related behavior analysis was conducted using video materials.

### 2.5 Statistical analysis

SPSS 26.0 and Mplus 8.3 were used for the statistical analysis. Continuous variables are shown as means and standard deviations, and categorical variables are presented as frequencies and percentages.

Baseline differences were analyzed by independent-samples *t*-tests and chi-square tests. Repeated-measures analysis of variance or linear mixed-effects models were used to assess the intervention effect, and group, time and group-by-time interactions were included as main effects.

Age, sex, family arts-education investment, parental education level, baseline piano experience, screen time, and exercise were all taken as potential covariates.

Pearson correlation analysis examined the relationships among the measured psychosocial outcomes, cross-cultural musical competence and cultural identity. A mediation and a serial mediation model were employed to test the proposed path linking the intervention, cross-cultural musical competence, cultural identity and the model-level psychosocial outcome construct. The label given to this analytic outcome construct in the original model output was “psychosocial wellbeing”; it was not an additional independently administered clinical scale.

The serial pathway was set up as follows: multicultural piano education → cross-cultural musical competence → cultural identity → psychosocial outcome construct.

The archived model documentation does not retain sufficient indicator-level information to determine whether the model-level outcome was specified as a latent factor, a standardized composite, or a single selected indicator without making *post-hoc* assumptions. Therefore, the revision will keep the originally estimated coefficients but explicitly state that this analysis is exploratory; no composite-scoring algorithm will be invented, and the construct will not be presented as a validated independent measure. Confirmatory replication should specify and report the measurement model, indicator loadings or scoring rules, direction coding and reliability.

Use the 5,000 bootstrap samples and a 95% confidence interval for the indirect effect. Missing questionnaire data were handled by multiple imputation, and full-information maximum likelihood estimation was used in other places. Set the significance level as *p* < 0.05.

### 2.6 Ethical considerations

The Ethics Committee of the institution has approved the research plan. Guardians provided written informed consent, and children provided age-appropriate assent to participate.

Participation was voluntary, and the children could leave at any time. Separate storage for personal data and reporting of only the aggregated results of the study were carried out.

## 3 Results

### 3.1 Participant flow and baseline characteristics

A total of 320 children were selected as the subjects and divided into the multicultural piano education group (*n* = 160) and the traditional piano education group (*n* = 160). After the intervention, 154 children in the experimental group and 152 children in the control group participated in all the assessments. Data for 150 and 147 participants were available at the 3-month follow-up, respectively. The entire follow-up rate was 0.928. The participant flow is as follows: Figure 2.

**Figure 2:**
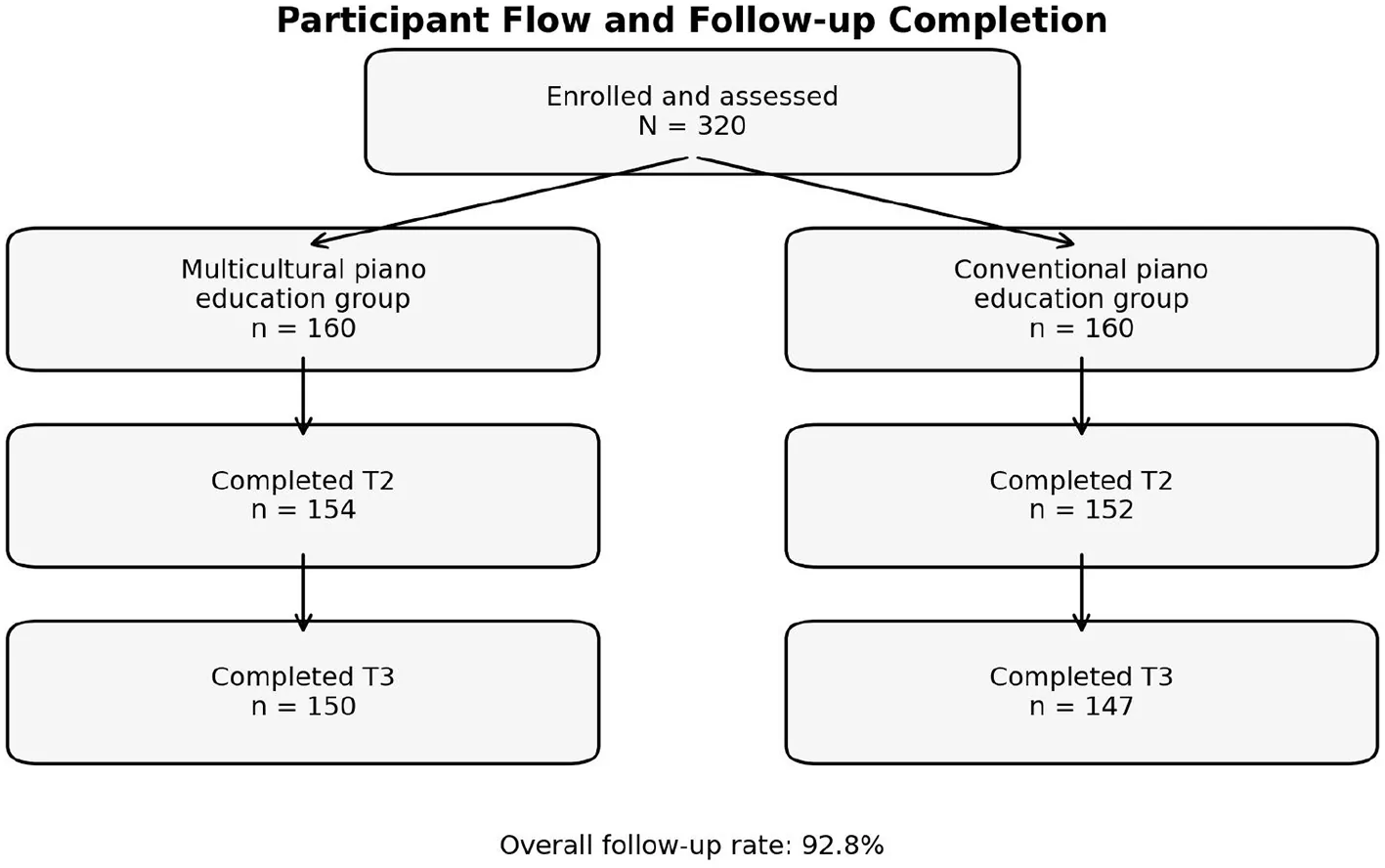
Participant flow and follow-up completion.

There were no significant baseline differences between the two groups in age, sex, place of residence, previous musical education, SCARED anxiety levels, social ability, cultural identity, or cross-cultural musical ability (all *p* > 0.05). The means of participation in the experimental group and the control group were 91.8 and 89.7%, respectively (Table 1).

### 3.2 Effects on psychosocial wellbeing

Significant group-by-time interactions were observed for the scores of SCARED anxiety, emotion-regulation, social competence, loneliness, self-esteem, psychological resilience, and heart-rate variability.

The experimental group reduced the SCARED anxiety scores more significantly than the control group. The mean SCARED scores in the intervention group and the control group after the intervention were 18.72 and 23.61, respectively; at baseline, they were 26.10 and 25.82. The interaction term was significant: *F*(2, 606) = 26.84, *p* < 0.001, and the partial eta-squared was 0.081. These values are interpreted as changes in scale scores, not as evidence that participants have met or no longer met the criteria for an anxiety disorder. Changes in the SCARED scores at different times are shown in Figure 3.

**Figure 3:**
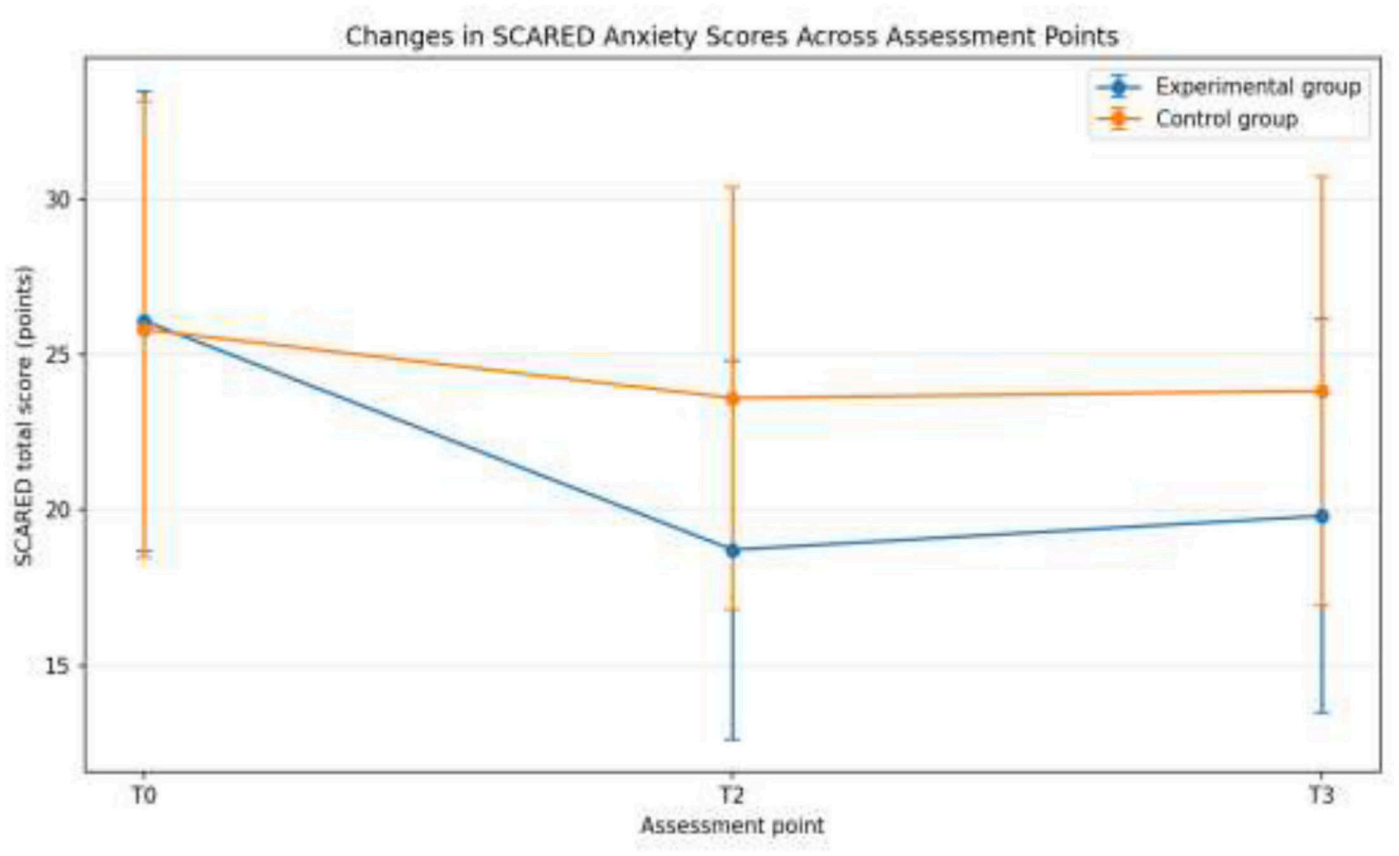
Changes in SCARED anxiety scores at different times.

Children in multicultural piano classes also showed better results in cognitive reappraisal, social skills, self-esteem, and emotional resilience. Loneliness scores dropped more substantially in the experimental group. Most of the improvements occurred by 3 months. The modification path of the main psychosocial outcomes is shown in Figure 4 and Table 4.

**Figure 4:**
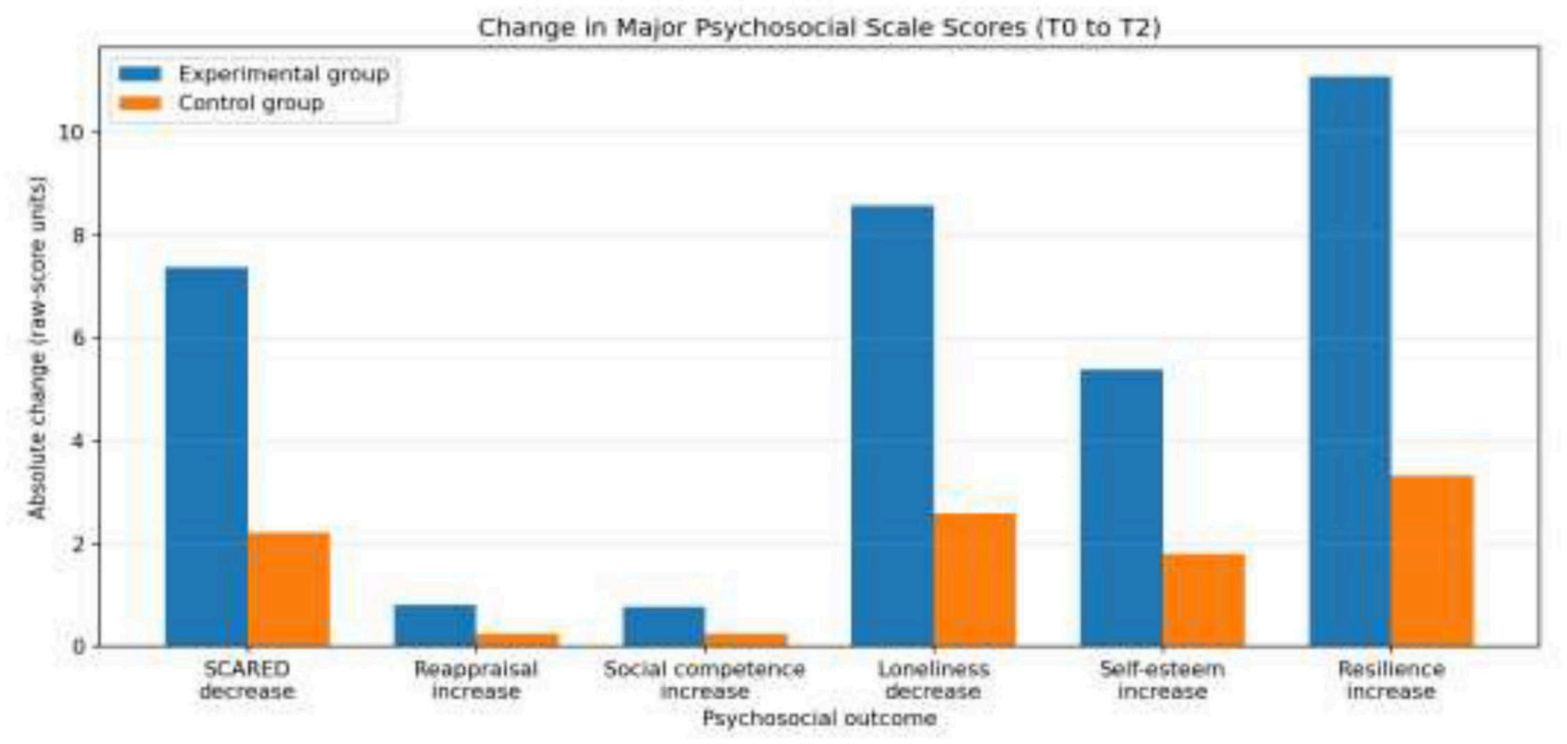
Absolute raw-score changes in the major psychosocial measures from baseline to post-intervention. The original score units of the measures have changed, so they cannot be directly compared.

**Table 4:** Changes in major psychosocial outcomes.

Outcome	Group	T0	T2	T3	Group × time *p*
SCARED anxiety score	Experimental	26.10 ± 7.42	18.72 ± 6.11	19.81 ± 6.35	< 0.001
	Control	25.82 ± 7.35	23.61 ± 6.82	23.84 ± 6.91	
Cognitive reappraisal	Experimental	3.02 ± 0.61	3.82 ± 0.57	3.74 ± 0.59	< 0.001
	Control	3.05 ± 0.63	3.29 ± 0.60	3.31 ± 0.62	
Social competence	Experimental	3.12 ± 0.49	3.89 ± 0.44	3.80 ± 0.47	< 0.001
	Control	3.10 ± 0.51	3.35 ± 0.48	3.36 ± 0.49	
Loneliness score	Experimental	39.82 ± 8.13	31.24 ± 7.01	32.42 ± 7.25	< 0.001
	Control	39.51 ± 8.05	36.91 ± 7.62	37.03 ± 7.71	
Self-esteem score	Experimental	25.42 ± 4.57	30.81 ± 4.21	30.12 ± 4.33	< 0.001
	Control	25.61 ± 4.48	27.42 ± 4.39	27.58 ± 4.45	
Resilience score	Experimental	58.62 ± 10.34	69.71 ± 9.42	68.93 ± 9.61	< 0.001
	Control	59.08 ± 10.17	62.41 ± 9.88	62.76 ± 9.95	

At T2, the resting RMSSD in the experimental group was 41.83 ± 11.07 ms and increased from 34.62 ± 10.18 ms, whereas in the control group, it rose only slightly to 36.91 ± 10.56 ms from 35.07 ± 10.42 ms. The group-by-time interaction was significant, F(2, 598) = 14.72, p < 0.001, partial η^2^ = 0.047.

### 3.3 Cross-cultural musical competence

Cross-cultural Musical Competence increased significantly in the experimental group. The accuracy of auditory discrimination at T2 was 78.9%, up from 51.8% at baseline, and in the control group, it only increased to about 52.1% by around 7.3%. The development path of cross-cultural musical competence is shown in Figure 5 below.

**Figure 5:**
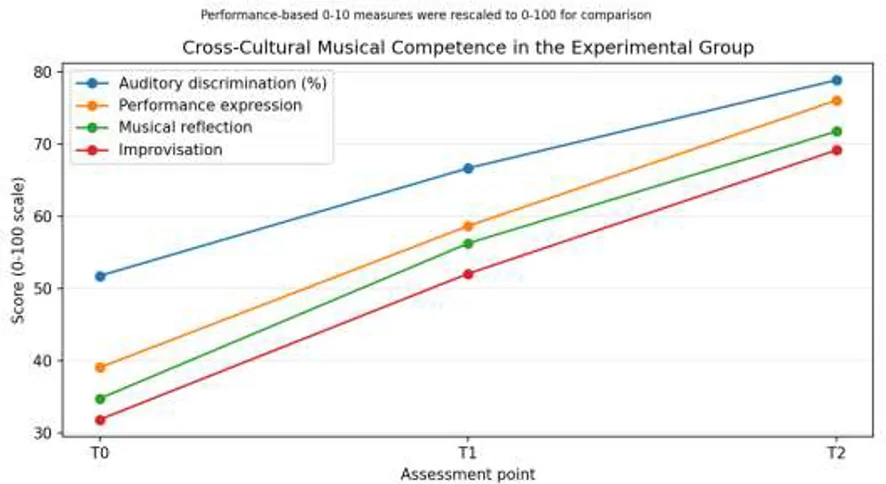
Changes in cross-cultural musical competence during the intervention.

The experimental group also showed higher post-intervention scores in culturally appropriate performance, musical reflection and multicultural improvisation (Table 5).

**Table 5:** Changes in cross-cultural musical competence.

Outcome	Group	T0	T1	T2	Interaction *p*
Auditory discrimination, %	Experimental	51.8 ± 11.7	66.7 ± 10.9	78.9 ± 9.8	< 0.001
	Control	52.1 ± 11.5	56.0 ± 11.1	59.4 ± 10.7	
Performance expression, 0–10	Experimental	3.91 ± 1.12	5.87 ± 1.08	7.61 ± 1.03	< 0.001
	Control	3.96 ± 1.09	4.51 ± 1.07	5.12 ± 1.11	
Musical reflection, 0–10	Experimental	3.48 ± 1.20	5.63 ± 1.16	7.18 ± 1.09	< 0.001
	Control	3.55 ± 1.18	4.17 ± 1.14	4.82 ± 1.17	
Multicultural improvisation, 0–10	Experimental	3.19 ± 1.14	5.21 ± 1.19	6.92 ± 1.15	< 0.001
	Control	3.25 ± 1.17	3.88 ± 1.13	4.41 ± 1.18	

The two blinded piano assessors had good inter-rater reliability, and the intraclass correlation coefficients were as high as 0.91.

### 3.4 Cultural identity

Local cultural identity, acceptance of other cultures, and bicultural integration have all increased.

At T2, the experimental group had higher local cultural identification than the control group (5.48 ± 0.68 vs. 4.72 ± 0.71, *p* < 0.001). The same difference was observed for the acceptance of other cultures (5.56 ± 0.65 vs. 4.49 ± 0.74, *p* < 0.001) and bicultural integration (5.37 ± 0.71 vs. 4.34 ± 0.76, *p* < 0.001). Differences in cultural identity dimensions after intervention are shown in Figure 6.

**Figure 6:**
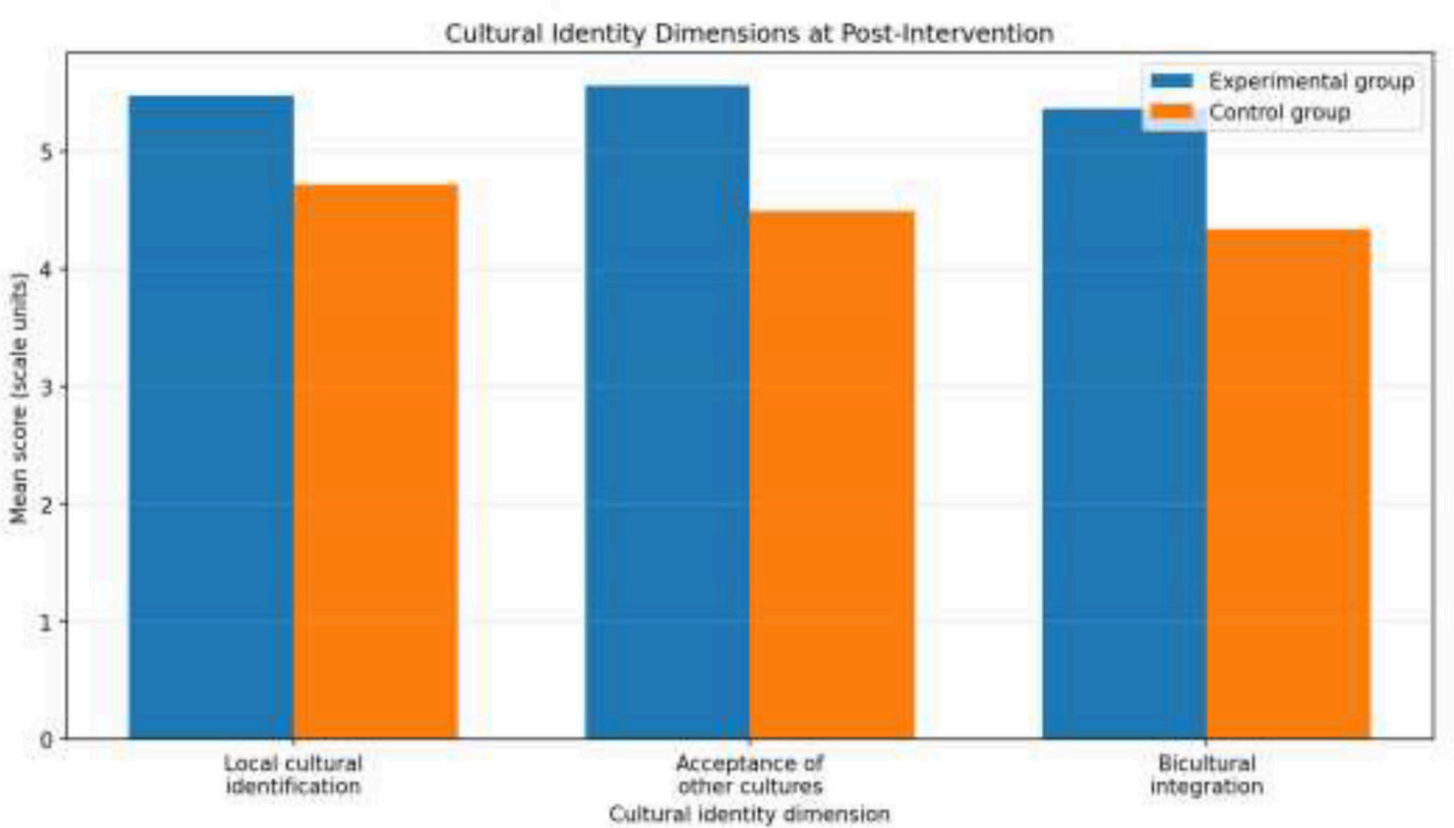
Cultural identity dimensions at post-intervention.

The above effects were still statistically significant at T3, and a slight decrease occurred after the intervention.

### 3.5 Mediation analysis

Structural equation modeling showed that multicultural piano education was positively associated with cross-cultural musical competence (β = 0.49, *p* < 0.001). Cross-cultural musical competence was positively correlated with cultural identity (β = 0.42, *p* < 0.001), and cultural identity was positively correlated with the model-level psychosocial outcome construct (β = 0.31, *p* < 0.001). Figure 7 is this serial path. As the archived model documentation does not permit the reconstruction of the outcome construct at the indicator level, these mediation results are interpreted as exploratory rather than as validation of a new psychosocial wellbeing scale.

**Figure 7:**
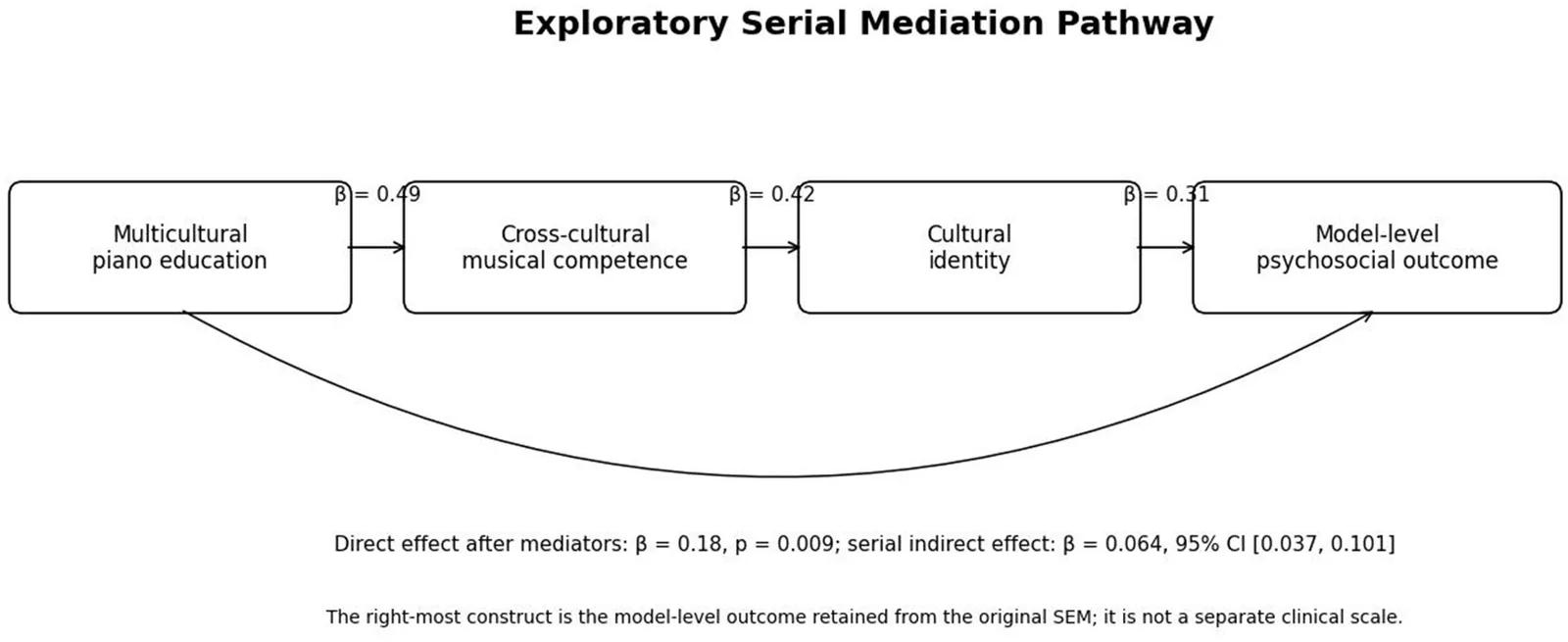
Exploratory serial mediation pathway from multicultural piano education to the model-level psychosocial outcome construct. The rightmost box is labeled “psychosocial wellbeing” in the original model output; it is an analytical outcome construct employed in the SEM rather than an independent clinical scale.

The total indirect effect of the intervention on the psychosocial outcome construct was positive and statistically significant [β = 0.23, 95% CI (0.16, 0.31)]. The serial indirect path *via* cross-cultural musical competence and cultural identity was also significant [β = 0.064, 95% CI (0.037, 0.101)].

Direct effect is still significant after adding the mediators (β = 0.18, *p* = 0.009), so partial mediation occurs. The model showed good fit: χ^2^/df = 2.08, CFI = 0.961, TLI = 0.949, RMSEA = 0.058, and SRMR = 0.043.

Baseline cultural identity did not significantly moderate the effect of intervention (β = −0.07, *p* = 0.180).

### 3.6 Behavioral outcomes and safety

Classroom observations showed that the children in the experimental group were more willing to speak and work with others in music activities than those in the control group. They also had fewer coordination errors and withdrawal behaviors by the end of the intervention.

No severe adverse events occurred. Temporary hand fatigue was reported by 15 children in the experimental group and 12 children in the control group. All cases were solved by rest and exercise. No one left due to serious harm in their body or mind.

## 4 Discussion

Investigate the effect of a 12-week multicultural piano education program on the psychosocial wellbeing, cross-cultural musical literacy and cultural identity of children aged 6–12. Compared with the group of children in traditional piano classes, the multicultural piano education group showed more favorable changes in SCARED anxiety scores, emotion-regulation scores, social competence, loneliness, self-esteem, psychological resilience, and heart-rate variability. Intervention also increased the sense of cross-cultural musical literacy and cultural identity. Mediation analysis further indicated that cross-cultural musical competence and cultural identity partially accounted for the relationship between multicultural piano education and the psychosocial outcome model.

### 4.1 Effects on psychosocial wellbeing

The reduction in SCARED anxiety scores may be due to the emotional expression and progressive organization of piano learning (Birmaher et al., 1997; Saarikallio and Erkkilä, 2007). Children were repeatedly given some achievable musical tasks and allowed to correct their mistakes, finish the performance, and feel successful. These experiences may have increased perceived competence and decreased anxiety-related ratings of failure or evaluation. Since the participants were not selected based on having anxiety disorders or high clinical risks, these results should be viewed as differences in questionnaire scores rather than as evidence of anxiety remission or treatment of anxiety psychopathology.

Intervention also included cooperative performance and musical discussion (Kirschner and Tomasello, 2010; Rabinowitch et al., 2013; Schellenberg et al., 2015). Such activities had to be done in a group, so children had to communicate and cooperate. These repeated interpersonal experiences may be responsible for the higher social-competence scores and lower loneliness scores of the multicultural group. Rather than individual piano practice, the multicultural curriculum focused more on cooperation and shared musical creation.

Higher self-esteem and psychological resilience scores may have resulted from successful performance experiences and positive feedback from teachers and peers (Bandura, 1977; Ryan and Deci, 2000). To learn the new music and culture, some people were reluctant. As the children gradually learned to do so, they probably gained some confidence in handling problems on their own.

An increase in resting RMSSD offers the first physiological support for more favorable autonomic regulation (Shaffer and Ginsberg, 2017; Task Force of the European Society of Cardiology and the North American Society of Pacing and Electrophysiology, 1996). It should not be taken as a direct or particular index of emotional regulation. HRV is affected by age, recent exercise, sleep, breathing, posture and measurement conditions; therefore, the physiological data should be interpreted cautiously and in combination with the results of questionnaires and behavior observations.

### 4.2 Effects on cross-cultural musical competence

The experimental group showed better improvements in auditory discrimination, culturally appropriate performance, musical reflection and multicultural improvisation. This is the expected result, given that the curriculum includes listening, performance, cultural explanation, comparison and creation.

The first two are hearing discrimination and expressing one's thoughts verbally. By repeatedly being exposed to all sorts of musical styles and expressions, children have come to know each other better. Cultural storytelling may have helped children connect the musical structure to their own history and society, so unfamiliar pieces were not treated as isolated technical problems.

Improvement in multicultural improvisation suggests that children could learn about the culture through music and apply it in their own ways. It may be that the learner has achieved a higher level of learning through this.

### 4.3 Effects on cultural identity

Multicultural piano education promoted local cultural awareness, openness to other cultures and bicultural integration (Benet-Martínez and Haritatos, 2005; Guan et al., 2023; Phinney, 1992). The increase in local cultural identification shows that exposure to foreign works has not made the children lose touch with their own culture. Comparisons of Chinese ethnic music with other musical cultures may have helped some people better understand the charm of their own music.

At the same time, the children were more open to learning new cultures. Cultural explanations, comparative listening and group discussions may have reduced the perception of foreign music as strange or distant. Children were led to learn about the culture of others through music and not judged by a single standard of beauty.

Strengthening bicultural integration shows that children can now experience the local and foreign musical cultures harmoniously. Based on the above analysis, multicultural education aims to foster both cultural confidence and intercultural openness simultaneously, and these are not in opposition.

### 4.4 Mediating roles of musical competence and cultural identity

In terms of the exploratory mediation model, cross-cultural musical competence and cultural identity partially mediated the effect of the intervention on the model-level psychosocial outcome construct. One possible reason is that children first developed a stronger sense of ability in learning and doing various cultures' music, thus feeling less anxious about encountering new cultures and gaining a sense of accomplishment. Partial mediation also indicates that other unobserved factors, such as teacher support, peer cooperation, practice quality and family involvement, may have been responsible for the observed effects.

Enhanced musical ability may have provided strong support for an all-encompassing cultural consciousness. Children who have learned both local and foreign music cultures may feel more culturally proud, curious and open-minded. The above identity-related changes may have increased emotional confidence and social adaptation, thus enhancing psychological wellbeing.

The rest of the direct effects are due to other reasons. Plausible reasons include teacher support, enjoyment, musical achievement, peer cooperation, increased attention and regular participation in organized educational activities. These variables were not directly included in the current model and will need to be considered separately in the future mechanism-oriented studies.

### 4.5 Practical implications

Therefore, multicultural content can be incorporated into piano classes without reducing the learning of piano techniques (Abril, 2006, 2013; Cain, 2015). Piano teachers can use culturally diverse works with background stories, comparative listening and group performances, and age-appropriate creative activities.

Rhythm imitation, storytelling, drawing and simplified repertoires are all suitable for young children. Older children can learn about culture through comparisons, improvisation, group performances and themed creations.

Schools and community arts organizations can also use multicultural piano education to integrate aesthetic education, cultural learning and psychosocial development in a low-risk manner. Provide better training for teachers to prevent a one-size-fits-all or superficial presentation of the culture.

### 4.6 Limitations

The following are some deficiencies. Firstly, the subjects were taken from a small number of schools, and thus the results might not be generalisable. Second, although this study conducted a 3-month follow-up, we do not know whether this effect will continue for a longer time.

Third, several outcomes relied on child self-report, and the sample included children as young as 6 years. The ERQ-CA was first applied to older children and adolescents; the standard RSES has mainly been collected from adolescents and adults; and the original CD-RISC was developed in adults. Developmental differences in reading comprehension, abstract self-evaluation and response styles may have affected the validity of the measurements for the youngest children. Therefore, the present study treats these results as continuous dimensional scores, avoids clinical cut-offs and diagnostic language, and does not infer treatment of psychopathology. In the future, use age-validated instruments and pre-determined assisted-administration protocols prospectively for younger children, along with parent, teacher, behavior, and physiological observations.

Fourth, the interpretation of HRV is limited by missing technical information on acquisition and preprocessing. Although the study report documents 5-min resting recordings and the use of SDNN and RMSSD, HRV is sensitive to the time of day, recent physical exercise, breathing and sleep, posture, device characteristics, and artifact-processing procedures (Shaffer and Ginsberg, 2017; Task Force of the European Society of Cardiology and the North American Society of Pacing and Electrophysiology, 1996). Therefore, the physiological findings should be regarded as supporting and extending the research to determine whether the intervention has modified emotional regulation directly.

Fifthly, complete blinding was not feasible because teachers and participants were aware of the teaching content. The performance assessors were blind, but expectancy effects may have still influenced the participation and self-reported data.

Sixth, the intervention had many parts, such as cultural narration, performance training, comparative appreciation and collaborative expression. Thus, the individual contributions of the components could not be identified.

Some Measurement Deficiencies Have Been Found. Some questionnaires were originally validated in age groups older than those of the youngest participants, and the archived records do not support the reconstruction of a 6–8 year-old administration mode or sample-specific internal-consistency coefficients. Independently validate the psychometric properties of a study-specific cultural identity index. In addition, the archived SEM documentation does not allow for a full reconstruction of the indicator-level specification of the model-level psychosocial outcome construct. Future research will specify age-appropriate administration procedures, retain item-level response and reliability data, standardize HRV acquisition, document observational coding reliability, and report the complete SEM measurement model.

## 5 Conclusion

Based on the above, this study proposes that a 12-week multicultural piano education program may be positively correlated with favorable psychosocial scale profiles, cross-cultural musical competence, cultural identity and resting HRV in children aged 6–12 years. Compared with traditional piano teaching, students in the multicultural curriculum had lower SCARED anxiety and loneliness scores and higher emotion-regulation, social-competence, self-esteem and psychological-resilience scores, as well as favorable changes in HRV. The above are the results of dimensional research and should not be regarded as a diagnosis or treatment of psychopathology.

Intervention also helped the children learn to recognize, perform, interpret and create works of music from different cultures. Improved local cultural consciousness and acceptance of other cultures, as well as bicultural integration. Exploratory mediation analysis showed that cross-cultural musical competence and cultural identity partially accounted for the path through which multicultural piano education related to the model-level psychosocial outcome construct; confirmatory studies using a pre-specified measurement model are required before making stronger mediation claims.

Based on the above results, integrating Chinese ethnic music and foreign works can be used to tell stories and compare cultures through piano performance and group singing. Therefore, multicultural piano education can be employed to integrate music education, intercultural learning and the psychological development of children in schools and community arts institutions.

Further research will employ multiple-center studies, validated cross-cultural assessment tools, extended follow-up periods, and objective behavior and physiological indicators to investigate the efficacy and underlying mechanisms of this intervention in greater detail.

## Data Availability

The raw data supporting the conclusions of this article will be made available by the authors, without undue reservation.

## References

[B1] Abril C. R. (2006). Music that represents culture: selecting music with integrity. Music Educat. J. 93, 38–45. doi: 10.1177/002743210609300122

[B2] Abril C. R. (2013). Toward a more culturally responsive general music classroom. Gen. Music Today 27, 6–11. doi: 10.1177/1048371313478946

[B3] Asher S. R. Hymel S. Renshaw P. D. (1984). Loneliness in children. Child Dev. 55, 1456–1464. doi: 10.2307/1130015

[B4] Bandura A. (1977). Self-efficacy: toward a unifying theory of behavioral change. Psychol. Rev. 84, 191–215. doi: 10.1037/0033-295X.84.2.191 847061

[B5] Benet-Martínez V. Haritatos J. (2005). Bicultural identity integration: components and psychosocial antecedents. J. Pers. 73, 1015–1050. doi: 10.1111/j.1467-6494.2005.00337.x 15958143

[B6] Birmaher B. Khetarpal S. Brent D. Cully M. Balach L. Kaufman J. . (1997). The Screen for Child Anxiety Related Emotional Disorders (SCARED): scale construction and psychometric characteristics. J. Am. Acad. Child Adolesc. Psychiatry 36, 545–553. doi: 10.1097/00004583-199704000-00018 9100430

[B7] Blasco-Magraner J. S. Bernabe-Valero G. Marín-Liébana P. Moret-Tatay C. (2021). Effects of the educational use of music on 3- to 12-year-old children's emotional development: a systematic review. Int. J. Environ. Res. Public Health 18:3668. doi: 10.3390/ijerph18073668 33915896 PMC8037606

[B8] Bortz G. Ilari B. Germano N. D. G. Jackowski A. P. Cogo-Moreira H. Lúcio P. S. (2025). The impact of music education on children's cognitive and socioemotional development: a quasi-experimental study in the Guri Program in Brazil. PLOS ONE 20:e0314355. doi: 10.1371/journal.pone.0314355 41100459 PMC12530532

[B9] Cain M. (2015). Musics of “the other”: Creating musical identities and overcoming cultural boundaries in Australian music education. Brit. J. Music Educ. 32, 71–86. doi: 10.1017/S0265051714000394

[B10] Connor K. M. Davidson J. R. T. (2003). Development of a new resilience scale: the Connor–Davidson Resilience Scale (CD-RISC). Depress. Anxiety 18, 76–82. doi: 10.1002/da.10113 12964174

[B11] Dingle G. A. Hodges J. Kunde A. (2016). Tuned In emotion regulation program using music listening: effectiveness for adolescents in educational settings. Front. Psychol. 7:859. doi: 10.3389/fpsyg.2016.00859 27375537 PMC4894878

[B12] Fu H. Tu J. (2023). Exploring the influence of national music lessons on subjective well-being, self-esteem, and national identity among university students: a case study from China. Front. Psychol. 14:1151007. doi: 10.3389/fpsyg.2023.1151007 37404590 PMC10316515

[B13] Gerry D. Unrau A. Trainor L. J. (2012). Active music classes in infancy enhance musical, communicative and social development. Dev. Sci. 15, 398–407. doi: 10.1111/j.1467-7687.2012.01142.x 22490179

[B14] Gross J. J. John O. P. (2003). Individual differences in two emotion regulation processes: implications for affect, relationships, and well-being. J. Pers. Soc. Psychol. 85, 348–362. doi: 10.1037/0022-3514.85.2.348 12916575

[B15] Guan T. Luo N. Matsunobu K. (2023). Nurturing student ethnic identity through culturally responsive music teaching in China. Int. J. Music Educ. 41, 598–615. doi: 10.1177/02557614221132550

[B16] Gullone E. Taffe J. (2012). The Emotion Regulation Questionnaire for Children and Adolescents (ERQ-CA): a psychometric evaluation. Psychol. Assess. 24, 409–417. doi: 10.1037/a0025777 22023559

[B17] Hallam S. (2010). The power of music: its impact on the intellectual, social and personal development of children and young people. Int. J. Music Educ. 28, 269–289. doi: 10.1177/0255761410370658

[B18] Jaschke A. C. Honing H. Scherder E. J. A. (2018). Longitudinal analysis of music education on executive functions in primary school children. Front. Neurosci. 12:103. doi: 10.3389/fnins.2018.00103 29541017 PMC5835523

[B19] Kirschner S. Tomasello M. (2010). Joint music making promotes prosocial behavior in 4-year-old children. Evol. Hum. Behav. 31, 354–364. doi: 10.1016/j.evolhumbehav.2010.04.004

[B20] Moreno S. Bialystok E. Barac R. Schellenberg E. G. Cepeda N. J. Chau T. (2011). Short-term music training enhances verbal intelligence and executive function. Psychol. Sci. 22, 1425–1433. doi: 10.1177/0956797611416999 21969312 PMC3449320

[B21] Nan Y. Liu L. Geiser E. Shu H. Gong C. C. Dong Q. . (2018). Piano training enhances the neural processing of pitch and improves speech perception in Mandarin-speaking children. Proc. Natl. Acad. Sci. USA. 115, E6630–E6639. doi: 10.1073/pnas.1808412115 29941577 PMC6048476

[B22] Phinney J. S. (1992). The multigroup ethnic identity measure: a new scale for use with diverse groups. J. Adolesc. Res. 7, 156–176. doi: 10.1177/074355489272003

[B23] Rabinowitch T. C. Cross I. Burnard P. (2013). Long-term musical group interaction has a positive influence on empathy in children. Psychol. Music 41, 484–498. doi: 10.1177/0305735612440609

[B24] Ryan R. M. Deci E. L. (2000). Self-determination theory and the facilitation of intrinsic motivation, social development, and wellbeing. Am. Psychol. 55, 68–78. doi: 10.1037/0003-066X.55.1.68 11392867

[B25] Saarikallio S. Erkkilä J. (2007). The role of music in adolescents' mood regulation. Psychol. Music 35, 88–109. doi: 10.1177/0305735607068889

[B26] Schellenberg E. G. Corrigall K. A. Dys S. P. Malti T. (2015). Group music training and children's prosocial skills. PLOS ONE 10:e0141449. doi: 10.1371/journal.pone.0141449 26506414 PMC4624672

[B27] Schmitt D. P. Allik J. (2005). Simultaneous administration of the Rosenberg Self-Esteem scale in 53 nations: exploring the universal and culture-specific features of global self-esteem. J. Pers. Soc. Psychol. 89, 623–642. doi: 10.1037/0022-3514.89.4.623 16287423

[B28] Shaffer F. Ginsberg J. P. (2017). An overview of heart rate variability metrics and norms. Front. Public Health 5:258. doi: 10.3389/fpubh.2017.00258 29034226 PMC5624990

[B29] Task Force of the European Society of Cardiology and the North American Society of Pacing and Electrophysiology (1996). Heart rate variability: standards of measurement, physiological interpretation, and clinical use. Circulation 93, 1043–1065. doi: 10.1161/01.CIR.93.5.1043 8598068

